# Liver transcriptomic networks reveal main biological processes associated with feed efficiency in beef cattle

**DOI:** 10.1186/s12864-015-2292-8

**Published:** 2015-12-18

**Authors:** Pamela A. Alexandre, Lisette J. A. Kogelman, Miguel H. A. Santana, Danielle Passarelli, Lidia H. Pulz, Paulo Fantinato-Neto, Paulo L. Silva, Paulo R. Leme, Ricardo F. Strefezzi, Luiz L. Coutinho, José B. S. Ferraz, Joanie P. Eler, Haja N. Kadarmideen, Heidge Fukumasu

**Affiliations:** Department of Veterinary Medicine, School of Animal Science and Food Engineering, University of Sao Paulo, Av. Duque de Caxias Norte, 225, Pirassununga, São Paulo 13635-900 Brazil; Department of Veterinary Clinical and Animal Sciences, Faculty of Health and Medical Sciences, University of Copenhagen, Copenhagen, Denmark; Department of Animal Sciences, School of Animal Science and Food Engineering, University of São Paulo, Pirassunung, Sao Paulo Brazil; Department of Animal Sciences, ESALQ, University of Sao Paulo, Piracicaba, Sao Paulo Brazil

**Keywords:** RNA-Seq, co-expression network, residual feed intake, residual intake and body weight gain, inflammation, lipid metabolism

## Abstract

**Background:**

The selection of beef cattle for feed efficiency (FE) traits is very important not only for productive and economic efficiency but also for reduced environmental impact of livestock. Considering that FE is multifactorial and expensive to measure, the aim of this study was to identify biological functions and regulatory genes associated with this phenotype.

**Results:**

Eight genes were differentially expressed between high and low feed efficient animals (HFE and LFE, respectively). Co-expression analyses identified 34 gene modules of which 4 were strongly associated with FE traits. They were mainly enriched for inflammatory response or inflammation-related terms. We also identified 463 differentially co-expressed genes which were functionally enriched for immune response and lipid metabolism. A total of 8 key regulators of gene expression profiles affecting FE were found. The LFE animals had higher feed intake and increased subcutaneous and visceral fat deposition. In addition, LFE animals showed higher levels of serum cholesterol and liver injury biomarker GGT. Histopathology of the liver showed higher percentage of periportal inflammation with mononuclear infiltrate.

**Conclusion:**

Liver transcriptomic network analysis coupled with other results demonstrated that LFE animals present altered lipid metabolism and increased hepatic periportal lesions associated with an inflammatory response composed mainly by mononuclear cells. We are now focusing to identify the causes of increased liver lesions in LFE animals.

**Electronic supplementary material:**

The online version of this article (doi:10.1186/s12864-015-2292-8) contains supplementary material, which is available to authorized users.

## Background

The growing demand for protein and energy to supply the expanding human population brings great concerns to light regarding both improvement of productivity and reduction of environmental impact of livestock [1]. Feed efficiency (FE) in beef cattle is an important trait from productive and economic point of view because it identifies animals that present less feed intake to produce the same amount of beef and feed is considered the most expensive input in beef production [2]. Moreover, selection of efficient animals can increase the sustainability of the production system, as the production of greenhouse gases from livestock accounts for 14.5 % of the total emitted by human [3] and the difference of emission between feed efficient and inefficient animals can reach 28 % [4].

There are several traits to estimate FE in beef cattle, for example by residual feed intake (RFI), a well-accepted measure that is calculated by the difference between observed and predicted feed intake based on average daily gain (ADG) and metabolic weight [5]. Because ADG is an independent variable in the regression that estimates predicted feed intake, RFI and ADG have no correlation. Thus, RFI can select efficient animals that might also be slow growing. The recently proposed residual intake and body weight gain (RIG) combines RFI and residual body weight gain to generate a measure that presents favorable correlations with both ADG and feed intake [6].

Despite its relevance, selection for feed efficiency in beef cattle is a challenge in breeding programs, as it is necessary to measure daily feed intake and body weight gain individually for at least 70 days [7]. A better comprehension of the biological mechanisms regulating feed efficiency is crucial to identify biomarkers that could differentiate efficient from inefficient animals in an accurate, cheaper and time-saving way. Some previous works had investigated FE through global gene expression profile of liver, a central organ of metabolism which is responsible for important functions, including metabolism of bilirubin, bile acids, carbohydrates, lipids, xenobiotics, protein synthesis and immunity [8]. Metabolism, together with feed intake, digestion, physical activity and thermoregulation are the main biological mechanisms supposed to regulate FE [9, 10].

In a study with Angus cattle (*Bos taurus*), groups of high and low FE were analyzed using microarray technique and identified 161 differently expressed genes in liver, most of them related to growth and cellular organization, cell signaling and proliferation, metabolism of xenobiotics, protein synthesis and metabolism of lipids and carbohydrates [11]. In a recently published study in this journal, RNA sequencing (RNAseq) was used to evaluate liver transcriptomic of Nellore steers (*Bos indicus*) and 112 annotated genes were identified as being differentially expressed between animals with high and low FE [12]. The authors identified xenobiotic metabolism, complement and coagulation cascades, NRF2-mediated oxidative stress, melatonin degradation and glutathione metabolism as overrepresented pathways. The latest study on hepatic transcriptome associated to feed efficiency was performed using taurine heifers and suggested that animals of high and low feed efficiency respond differently to hepatic proinflammatory stimulus [13]. Despite the given indications of the mechanisms involved in regulation of feed efficiency, the analysis of differential expression (DE) performed by the aforementioned studies does not explain gene-to-gene interactions or the behavior of regulatory genes in a complex system, because when comparing a gene with itself in different conditions the context of this expression can be lost [14].

Gene expression network approaches are based on the fact that genes and their products can interact with each other by complex relationships, so the effect of a change in the behavior of one gene can be propagated through the interactions and affect other genes [15]. Network studies applied to transcriptomic data reveal more complex transcriptional regulation than DE by detecting sets of highly co-expressed genes (modules) [16, 17] that orchestrate complex traits. This approach can be particularly useful to reveal master regulatory or hub genes, since hub genes of modules are expected to play a critical role in regulating the expression of several dozens of other genes in the module and can in fact be used as biomarkers. This approach has been successfully applied in sheep to detect biomarkers for intestinal nematode resistance [18], wool growth [19] and in pig models for human obesity [17].

In the present study, we performed DE and co-expression network analysis using next generation sequencing based transcriptomics data (RNAseq) from liver biopsy samples of Nellore cattle with high and low feed efficiency to reveal biological functions and regulatory genes associated with this phenotype. To our knowledge, this is the first study using network methods to study functional genomics of feed efficiency in beef cattle and a step ahead to understand this economically important trait.

## Results

### Characterization of feed efficiency groups

We performed a 70-d feeding trial on 98 Nellore bulls and for each animal we obtained the initial body weight (BWi), final BW (BWf), dry matter intake (DMI), average daily gain (ADG), feed conversion ratio (FCR), residual feed intake (RFI), residual body weight gain (RWG) and residual intake and body weight gain (RIG). A summary of the phenotypic traits can be found in Additional file 1: Table S1. Two animals were removed from the analysis due to very low ADG. There was no significant sire effect on RFI and RIG and the phenotypic correlation between the two traits was −0.97 (*P* ≤ 0.05). The difference between the high feed efficiency (HFE, *n* = 20) and low feed efficiency (LFE, *n* = 20) groups, defined by RIG, for all phenotypic measures taken during the feeding trial and slaughter can be seen in Table 1. Significant difference was observed for all FE traits (FCR, RFI, RWG and RIG) and for DMI, which was higher in animals of LFE. There was also a significant difference for final and gain of back fat thickness (BFT) and for final and gain of rump fat thickness (RFT) in the period (*P* ≤ 0.05), which were higher in LFE group.

**Table 1:** Summary of phenotypic traits. Mean of the groups of high feed efficiency (HFE, *n* = 20) and low feed efficiency (LFE, *n* = 20) and P-value of the difference between groups for phenotypic traits measured during the feeding trial and slaughter

Trait	HFE mean	LFE mean	*P*-value
BWi (kg) ^♦^	403.10 ± 35.6	409.50 ± 23.0	0.50
BWf (kg)^○^	542.10 ± 46.9	533.90 ± 25.2	0.47
*DMI (kg/d)* ^*♦*^	*9.99 ± 1.3*	*12.00 ± 0.7*	1.03x10-6*
ADG (kg/d) ^♦^	1.97 ± 0.5	1.76 ± 0.2	0.06
*FCR* ^*♦*^	*5.22 ± 0.8*	*6.90 ± 0.8*	2.84x10-8*
*RFI (kg/d)* ^*○*^	*−1.14 ± 0.4*	*1.24 ± 0.5*	6.79x10-8*
*RWG (kg/d)* ^*♦*^	*0.27 ± 0.3*	*−0.29 ± 0.2*	3.00x10-9*
*RIG* ^*○*^	*1.40 ± 0.4*	*−1.53 ± 0.6*	6.77x10-8*
REAi (cm^2^) ^*♦*^	67.51 ± 5.5	65.95 ± 5.3	0.36
REAf (cm^2^) ^*♦*^	83.49 ± 6.9	83.12 ± 6.0	0.85
REAg (cm^2^) ^*♦*^	15.98 ± 8.9	17.18 ± 6.4	0.63
BFTi (mm) ^*♦*^	1.18 ± 1.0	1.64 ± 1.2	0.19
*BFTf (mm)* ^*♦*^	*3.99 ± 1.9*	*5.78 ± 1.4*	*1.9x10* ^*−3*^ *
*BFTg (mm)* ^*♦*^	*2.81 ± 2.0*	*4.15 ± 1.4*	0.02*
RFTi (mm) ^*♦*^	2.78 ± 1.4	3.73 ± 1.9	0.08
*RFTf (mm)* ^*♦*^	*5.76 ± 2.4*	*8.19 ± 2.2*	*1.8x10* ^*−3*^ *
*RFTg (mm)* ^*♦*^	*2.99 ± 1.9*	*4.47 ± 1.6*	0.01*
LW (kg) ^*♦*^	6.00 ± 0.59	5.78 ± 0.54	0.48
CY (%)	60.11 ± 1.6	60.23 ± 1.6	0.83
*PFW (kg)* ^*♦*^	*4.44 ± 1.1*	*5.57 ± 1.3*	*5.9x10* ^*−3*^ *
*KFW (kg)* ^*○*^	*4.45 ± 1.1*	*5.63 ± 1.6*	*7.3x10* ^*−3*^ *

BWi, initial body weight; BWF, final body weight; DMI, dry matter intake; ADG, average daily gain; FCR, feed conversion ratio; RFI, residual feed intake; RWG, residual body weight gain; RIG, residual intake and body weight gain; REAi, initial rib eye area; REAf, final rib eye area; REAg, gain of rib eye area; BFTi, initial back fat thickness; BFTf, final back fat thickness; BFTg, gain of back fat thickness; RFTi, initial rump fat thickness; RFTf, final rump fat thickness; RFTg, gain of rump fat thickness; LW, liver weight; CY, carcass yield; PFW, pelvic fat weight; KFW, kidney fat weight

*P ≤ 0.05

^♦^Student’s t-test

^○^Mann–Whitney-Wilcoxon test

### Differentially expressed genes in the liver of HFE and LFE animals

A total of 11,361 genes were detected and tested for differential expression in the liver samples and 8 of them were differentially expressed (DE) between HFE and LFE (*P* ≤ 0.1) (Table 2). Respectively, seven transcripts (*SOD3, RHOB, mir-2904-3, ENSBTAG00000038430, CYP2E1, GADD45G* and *FASN*) and one transcript (*NR0B2*) were up and down regulated in the LFE group compared to the HFE group.

**Table 2:** Differentially expressed genes

GeneName	logFC	Pvalue	Padj
NR0B2	−1.4410	1.14x10^−08^	0.0001
SOD3	1.9112	1.68 x10^−07^	0.0010
RHOB	0.8498	8.11 x10^−07^	0.0031
bta-mir-2904-3	3.6411	5.42 x10^−06^	0.0154
ENSBTAG00000038430	1.8639	1.39 x10^−05^	0.0316
CYP2E1	3.1742	2.65 x10^−05^	0.0501
GADD45G	1.4682	3.75 x10^−05^	0.0609
FASN	1.3200	4.61 x10^−05^	0.0654

LogFC, Log2 (LFE CPM/ HFE CPM); HFE, High Feed Efficiency Group; LFE, Low Feed Efficiency Group; CPM, Counts Per Million

### Co-expressed genes and regulators

**Fig. 1:**
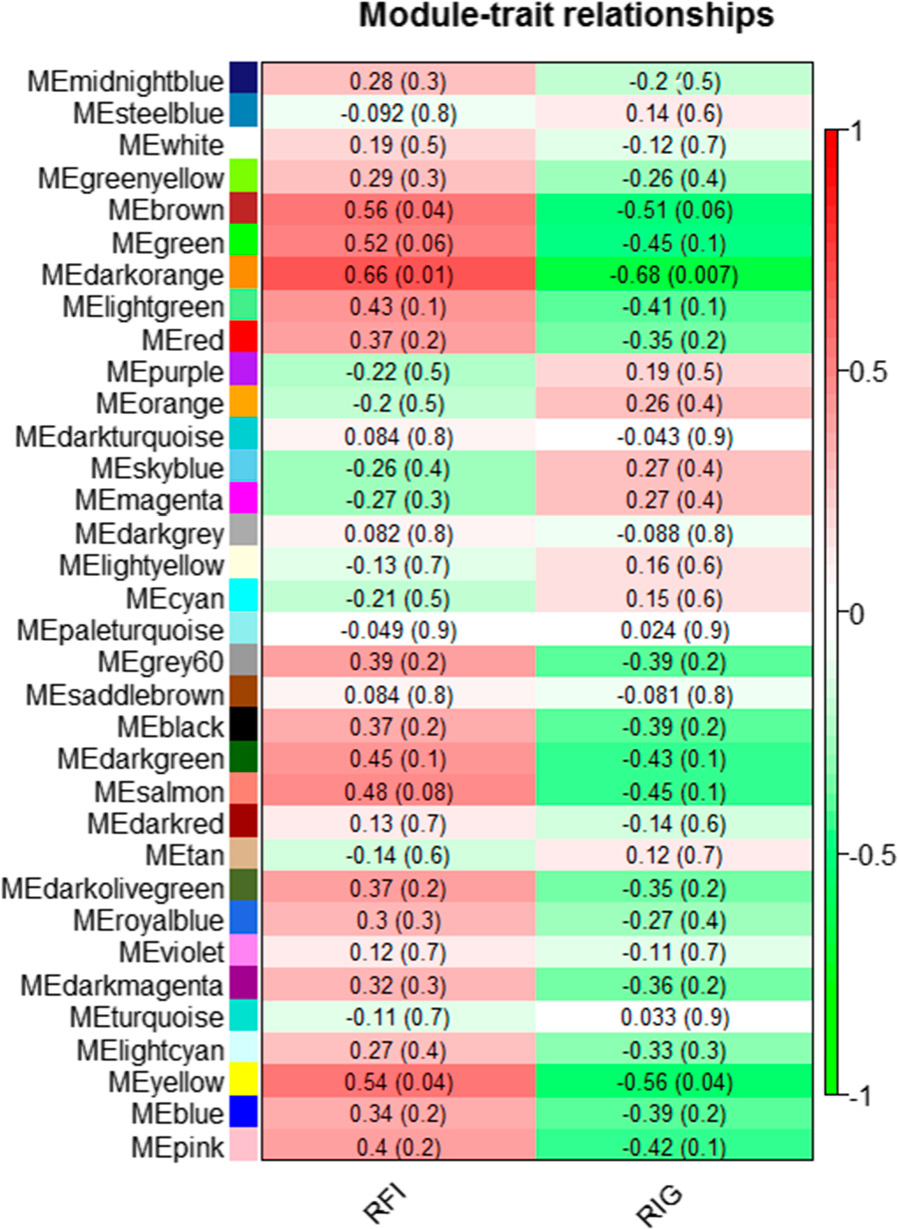
Correlations between module eigengene (ME) and FE traits - residual feed intake (RFI) and residual intake and body weight gain (RIG)

Using the WGCNA approach we identified 34 modules of co-expressed and highly interconnected genes and assigned different color names to each module (Fig. 1). Gene members of the same module are supposed to work cooperatively in related pathways or to be under control of a common set of transcription factor. Therefore, we tested the correlation of the modules eigengene with RFI and RIG to identify modules potentially representing biological mechanisms involved in feed efficiency regulation (Fig. 1). Four modules, *Brown, Green, Dark-orange* and *Yellow*, showed positive correlations with RFI and negative correlations with RIG, according to the established threshold (see Methods).

The *Brown* module included 94 unique genes after filtering for module membership (Additional file 1: Table S2) and those genes were used for functional enrichment. The functional enrichment showed significant results for oxidation-reduction process, regulation of monocyte differentiation and positive regulation of interleukin-8 biosynthetic process (Padj ≤ 0.1; Additional file 2). *Green* module included 55 unique genes (Additional file 3) and presented functional enrichment for protein targeting to mitochondria (*P* ≤ 0.05). *Dark-orange* module included 32 unique genes (Additional file 4) and presented functional enrichment for negative regulation of cell cycle (Padj ≤ 0.1). Finally the *Yellow* module included 90 unique genes (Additional file 5) and presented functional enrichment for many GO terms which are mostly related to gene expression, ribosomal biogenesis and protein translation; migration, proliferation and differentiation of T lymphocytes; and phagocytosis, but terms such as response to stress, regulation of intrinsic apoptotic signaling pathway and negative regulation of lipid transport also appeared.

In order to identify regulator genes for FE we used Lemon-Tree software suit which generated 115 clusters of co-expressed genes and assigned regulator genes to them. Three clusters were found to partially overlap with the previously identified *Brown, Green* and *Yellow* modules. *Cluster 7* presented 28 genes, 25 of them also belonging to the *Brown* module (WGCNA) and the other 3 belonging to *Dark-orange* and *Green* modules. Five regulators were assigned to this cluster, *HPCAL* (probabilistic score = 7.56, *P* ≤ 0.05), *SLC9A1* (probabilistic score = 9.24, *P* ≤ 0.05), *EZH2* (probabilistic score = 8.58, *P* ≤ 0.05), *DOLK* (probabilistic score = 9.32, *P* ≤ 0.05) and *PPP3CB* (probabilistic score = 10.87, *P* ≤ 0.05). Similarly, *Cluster 1* presented 33 genes, 29 of them also belonging to *Green* module (WGCNA). Two regulators were assigned to this cluster, *PGK1* (probabilistic score = 9.24, P ≤ 0.05) and *MVP* (probabilistic score = 9.24, *P* ≤ 0.05). At last, *Cluster 8* presented 25 genes, 22 of them also belonging to the *Yellow* module (WGCNA) and *RAMP3* was assigned as regulator of this module (probabilistic score = 5.81, *P* ≤ 0.05).

### Differentially co-expressed genes between HFE and LFE groups

We detected 463 differentially co-expressed genes between HFE and LFE groups (*|*K_diff_| > 0.6). Of those, 452 were highly connected in HFE and lowly connected in LFE, while 11 genes were highly connected in LFE and lowly connected in HFE (Additional file 6). Functional enrichment analysis of GO terms of all 463 genes showed that differentially co-expressed genes are related to immune/inflammatory response (macrophage and monocyte chemotaxis, antigen processing and presentation via MHC class II, response to other organism, response to stress (MAPK, JNK, ERK cascades), angiogenesis, cellular response to amino acid stimulus, regulation of phosphatidylinositol-3-kinase activity and fatty acid metabolic process (Padj ≤ 0.05).

### Investigation of liver expression profile results

In order to investigate the results indicated by liver transcriptome analysis of HFE and LFE groups, we performed serum biochemistry and liver histopathology. No significant difference was observed for all serum parameters (total cholesterol, triglycerides, globulins, AST, ALT, GGT, ALP, albumin and total protein) at the beginning of the feeding trial. However, at the end of the experiment, the LFE group presented increased serum cholesterol and GGT (*P* ≤ 0.05), both above the reference values for Nellore cattle [20] (Table 3). Thus, we aimed to evaluate the levels of serum cholesterol and GGT through the entire period of the feeding trial and we found significant differences (*P* ≤ 0.05) for serum cholesterol from day 14 until the last day (i.e. day 70). For serum GGT there was no significant difference until day 56, but there was a significant difference on day 70 (*P* ≤ 0.05). Even though not statistically significant (*P* > 0.05), it was possible to note the LFE group had increased serum GGT during the entire feeding trial.

**Table 3:** Summary of serum biochemistry analysis. Mean of serum parameters of groups of high feed efficiency (HFE, *n* = 20) and low feed efficiency (LFE, *n* = 20) on the first (day 0) and the last (day 70) day of feeding trial and p-value of the difference between the groups

Day	Parameter	Reference^§^	HFE mean	LFE mean	*P*-value
0	Cholesterol (mg/dL) ^♦^	80–120	112.33 ± 20.6	122.22 ± 25.9	0.1904
	Triglycerides (mg/dL)^○^	0–14	14.51 ± 5.6	12.62 ± 5.2	0.1675
	Globulin (g/dL)^○^	3.00–3.48	3.71 ± 0.5	3.51 ± 0.4	0.1045
	AST (U/L)^○^	78–132	82.25 ± 16.9	78.50 ± 42.8	0.5606
	ALT (U/L)^○^	11–40	20.25 ± 6.8	21.20 ± 5.0	0.6058
	GGT (U/L)^♦^	6.1–17.4	17.35 ± 5.1	18.15 ± 4.7	0.6084
	ALP (U/L)^○^	0–488	308.25 ± 89.4	322.35 ± 115.7	0.4818
	Albumin (g/dL)^♦^	3.03–3.55	2.78 ± 0.2	2.82 ± 0.2	0.4793
	Protein (g/dL)^○^	6.74–7.46	6.49 ± 0.5	6.33 ± 0.4	0.1894
70	Cholesterol (mg/dL)^♦^	*80–120*	*123.15 ± 26.5*	*147.45 ± 17.7*	0.0017*
	Triglycerides (mg/dL)^○^	0–14	11.61 ± 4.6	12.63 ± 3.4	0.6455
	Globulins (g/dL)^○^	3.00–3.48	3.41 ± 0.4	3.41 ± 0.3	0.5074
	AST (U/L)^○^	78–132	86.50 ± 12.6	82.30 ± 13.7	0.2179
	ALT (U/L)^○^	11–40	29.05 ± 5.2	28.45 ± 3.5	0.3149
	GGT (U/L)^○^	*6.1–17.4*	*15.25 ± 5.2*	*18.95 ± 3.3*	0.0150*
	ALP (U/L)^♦^	0–488	443.30 ± 96.8	382.30 ± 143.2	0.6560
	Albumin (g/dL)^○^	3.03–3.55	3.03 ± 0.2	3.07 ± 0.1	0.2284
	Protein (g/dL)^○^	6.74–7.46	6.44 ± 0.4	6.48 ± 0.3	0.9892

HFE, High Feed Efficiency Group; LFE, Low Feed Efficiency Group; AST, Aspartate Aminotransferase; ALT, Alanine Aminotransferase; GGT, Gamma-glutamyl Transpeptidase; ALP, Alkaline Phosphatase

*P ≤ 0.05

^§^KANEKO; HARVEY; BRUSS, 2008

^♦^Student’s t-test

^○^Mann–Whitney-Wilcoxon test

**Fig. 2:**
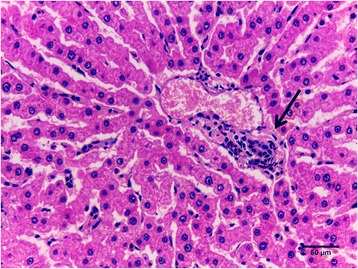
Liver histology. Periductal mononuclear infiltrate (arrow). HE, 400X

Next we performed liver histopathological analysis and all evaluated animals from both groups showed mononuclear infiltrate in the portal triad, especially in the periductal region. We observed a higher presence of this type of lesion on LFE animals (HFE: 81.76 ± 4.8 %; LFE: 91.70 ± 2.0 %; *P* = 0.1) (Fig. 2).

## Discussion

In this study, we determined possible mechanisms influencing feed efficiency in Nellore steers on feedlot, using liver transcriptomic network approaches coupled with serum biochemistry and liver histopathology. We observed that both HFE and LFE animals performed similarly for body weight gain, carcass yield and loin eye area, indicating that muscle deposition was similar between groups. We also observed that LFE animals had higher feed intake and greater deposition of subcutaneous and visceral fat which corroborates previous investigations [21–23]. However, the key find of this study was the enrichment of transcriptomic networks for inflammatory response in the liver of LFE animals, as well as other related terms as regulation of monocyte differentiation, process and migration; proliferation and differentiation of T lymphocytes, positive regulation of interleukin-8 biosynthesis, cell death through apoptosis, antigen presentation via MHC class II and response to other organisms. This inflammation proved by liver histopathology as periportal mononuclear infiltrate was a response to liver injury showed by increased serum GGT (a biomarker of liver injury). Evidence of association between another biomarker of liver injury, Aspartate aminotransferase (AST) and LFE animals was also previously reported in the literature [24]. Thus, the main question arising from this point on is: what is the cause of increased periportal liver lesions in LFE animals? The results found here led us to two main hypotheses: increased lipogenesis and/or higher bacterial infection in the liver, both being discussed below.

Consistently with higher feed intake, greater fat deposition and elevated level of serum cholesterol, LFE animals presented up regulated expression of Fatty Acid Synthase (*FASN*), which codes an enzyme that catalyze the synthesis of saturated fatty acids and regulate the lipid metabolism [25]. In ruminants, the lipogenesis primarily occurs in adipose tissues; however, it also occurs with limited capacity in the liver [26]. Other evidences of altered lipid metabolism between HFE and LFE groups were found in co-expression analysis. The positive correlation of the four identified co-expression modules with RFI and negative correlation with RIG shows that the lower the FE, the higher the general expression of the modules (module eigengene) and thus, the functions related to those modules are up regulated in the LFE group. Considering that, it is interesting to notice that *Yellow* module presented functional enrichment for regulation of lipid transport and *Green* module to protein targeting to mitochondria. This could be related to the first step of lipid metabolism which is the transport of fatty acids to the mitochondrion for β-oxidation [27]. Moreover, differentially co-expressed genes were also functionally enriched for fatty acid metabolic process. All together, these results indicated different metabolism of lipids in the liver of high and low FE animals.

Another interesting up regulated gene in LFE animals was the *CYP2E1*. Cytochrome P450 genes code for liver enzymes involved in maintaining the homeostasis of lipids (cholesterol, vitamin D metabolism and bile acids), compounds of endogenous decontamination processes (bile acids) and xenobiotics such as drugs and pesticides [28]; some CYPs were already related to FE in previous liver transcriptome analysis of FE in cattle [11, 12]. In humans and rodents there is a vast literature discussing the diverse functions of these enzymes, including in obesity [29, 30], but in domestic animals studies are scant [31]. The mechanism by which *CYP2E1* expression increases in human obesity are still controversial, but evidence suggests a relationship with insulin resistance which may have genetic causes or be connected to the supersaturation of energy molecules in the body (fatty acids, glycerol, glucose and acetyl CoA) [32]. Under conditions of elevated levels of fatty acids, the potential for uncoupled NADPH oxidation becomes more probable which results in increased oxygen-derived radicals and hydrogen peroxide [32]. Thus *CYP2E1* is an enzyme that generates reactive oxygen species (ROS) and reactive metabolites [32]. The elevation of ROS could explain the up regulation of *SOD3* in LFE animals. *SOD3* is a member of a protein family of antioxidant enzymes that catalyze the dismutation of two superoxide radicals into hydrogen peroxide and oxygen [33]. The elevated levels of *SOD3* expression could be a response to oxidative stress caused by the augmented lipid metabolism in the liver. This possibility became even stronger with the functional enrichment of *Brown* module for oxidation-reduction process, as differences in liver mitochondrial metabolism with higher ROS production in LFE animals as already reported on literature for pigs and broilers [34–38]. Other evidences of higher oxidative stress and expression of antioxidant enzymes in the liver of LFE cattle were also previously reported in other studies [11–13, 39]. Therefore, the hypothesis of LFE animals having increased lipid metabolism inducing an altered antioxidant response due to higher feed intake seems plausible.

On the other hand, it is interesting to notice that together with the enrichment for oxidation-reduction process, the *Brown* module is also enriched for regulation of monocyte differentiation and positive regulation of interleukin-8 biosynthesis, an important mediator of the immune reaction in the innate immune system response [40]. The fact that these two processes are present in the same module suggests a strong relationship between them – immune response can be an outcome of oxidative stress but also oxidative stress can be an outcome of immune response [41] and considering that, we will further discuss other possible causes of inflammation in liver.

GO terms associated with inflammation, immune response, migration and proliferation of T cells and response to stress are the most overrepresented both in *Yellow* module and in differentially co-expressed genes. The T lymphocytes are a class of leukocytes related to cell immunity [8] and when activated, molecules such as cytokines, adhesion molecules and prostaglandins are widely synthesized [8], which explains the fact that the *Yellow* module is also strongly related to translation of proteins. It should be considered that when analyzing liver tissue, not only one but several cells types have their expression levels captured. Thus, in case of hepatic inflammatory condition, the expression of immune cells that migrated to the affected area would also be considered in addition to the expression of hepatocytes, endothelial cells, stellate cells and Kupffer cells. Cytokines are important molecules for intracellular communication in the immune system because they bind to receptors on cell surface and activate intracellular signaling networks. An important component of these signaling networks is *GADD45* proteins which are especially well characterized in T cell [42]. That is probably why gene *GADD45G* is up regulated in LFE group. Another up regulated gene in LFE animals and can be associated with inflammatory response is *RHOB*, a tumor suppressor that is associated to cell adhesion, motility, proliferation and survival [43]. It was also demonstrated a proinflammatory activity of this gene in induced inflammation [43]. The only down regulated gene in LFE group is *NR0B2*, a member of the nuclear receptor family of intracellular transcription factors [44]. *NR0B2* inhibits numerous nuclear receptors and transcription factors in metabolic pathways such as bile acid synthesis, cholesterol and lipid metabolism, glucose metabolism, and energy homeostasis [45]. In addition, it was demonstrated that this gene have a role as an intrinsic endogenous regulator of homeostasis of the innate immune system by negative regulating inflammatory signaling [44] and that free fatty acids can repress its activation [46].

Voy and Aronow (2009) [47] used a systems genetic approach to analyze publicly available data from Shockley et al. (2009) [48] and showed that higher levels of cholesterol in mice are favorably correlated with inflammation, immunity and response to xenobiotic substances. In humans, higher serum cholesterol is associated with obesity [49, 50] and several reviews showed that overweight has a noticeable effect on the body's immune response leading to increased susceptibility to infections, although aspects of the association between obesity and immunity remain unclear [51–53]. The close proximity of cells responsible for metabolism and immunity in organs such as the liver and adipose tissue make close the relationship between these two biological processes [54].

As showed above, RNA-seq analysis is an exploratory approach that generates hypotheses to be further investigated by other assays. Thus, we showed that feed efficiency in beef cattle is associated with the expression of genes related to lipid metabolism but also hepatic inflammation. There is an important interplay between high fat deposition (obesity) with susceptibility to inflammation as demonstrated by several studies in humans [51–53] but at this point, one could argue that there could be other causes of liver inflammation in feedlot cattle, especially considering that the change of diet from pasture to high concentrate (corn and soy) is a stressful challenge to the animals which could lead to acidosis and even ruminitis [55–57]. Moreover, although acidosis usually occurs in the adaptation period to high energy diets, it can continue during all the feedlot period [55]. Nagaraja and Lechtenberg (2007) [56] argued that there is a higher prevalence of liver abscesses caused by bacteria from rumen in males than in females and this difference is related to higher feed intake observed in males. The same goes for Holstein animals breed for beef production, which also have higher feed intake and increased prevalence of liver abscesses when compared to beef breeds [56]. Considering that LFE animals presented higher feed intake during all the feeding trial, one might speculate that they could have also increased bacterial infection in the liver as demonstrated by more focal periportal lesions, since ruminitis can increase the contamination of mesenteric blood which enters the liver by portal artery. Bacterial infection could cause a reduction or even interruption of the bile flow (cholestasis), leading to the accumulation of bile acids and inducing cell death visualized by the increased serum GGT in LFE animals. It is important to remember that regulation of intrinsic apoptotic signaling pathway was one of the enriched terms in *Yellow* module and some of the terms enriched in differential co-expression, as antigen processing and presentation via MHC class II and response to other organisms, suggest that altered lipid metabolism is not the only possible cause of liver inflammation, but may predispose LFE animals to infection.

A recent study of differentially expressed genes in the liver of HFE and LFE taurine heifers showed that 5 of 7 differentially expressed genes were involved with innate immunity and were up regulated in LFE animals [13]. The authors hypothesized that HFE animals spend less energy to combat systemic inflammation because they have stronger or healthier hepatic innate immunity which results in better detoxification of endotoxins and bacterial products and, therefore, leave more energy available for growth and muscle deposition. Our results partially corroborate with this idea since we showed pronounced inflammatory response and altered lipid metabolism in the liver of LFE animals. However, we are more prone to believe that increased feed intake induced these alterations in the liver, since LFE animals eat more since the beginning of the feedlot period and the liver injury biomarker GGT was increased only in later times. Thus, low feed efficiency is probably not caused by intrinsic increased susceptibility to inflammation, but the latter is possibly an outcome of increased feed intake as is increased lipid metabolism.

Finally, the regulatory genes pointed in our study are centrals for the hepatic mechanisms involved in feed efficiency. Those genes need to be further evaluated and validated in a larger population but they are interesting potential biomarkers of feed efficiency in beef cattle. For example, *EZH2* is associated with hepatic homeostasis and regeneration [58] and its down-regulation causes the hepatocytes to become more susceptible to lipid accumulation and inflammation [59]; *DOLK* was demonstrated to be increased at least twofold as a result of inflammation [60]; and global *PPP3CB* knockout mice revealed phenotype related to diminished fat mass, protection from body weight gain and alterations in food intake, feed efficiency and energy expenditure [61].

## Conclusion

Animals with low feed efficiency present hepatic transcriptome associated with pronounced inflammation and lipid metabolism. Histopathology confirmed RNA-seq data since LFE animals have more liver periportal lesions associated with an inflammatory response composed mainly by mononuclear cells. Serum biomarker GGT confirmed increased liver lesions in LFE animals. Based on these results, in addition to information provided by literature, we were able to propose the hypothesis that the hepatic lesions in LFE animals occurred because of the stress generated by altered lipid metabolism and/or due to increased bacterial infection due to higher feed intake. At this point, it is hard to define if these two possibilities are associated to each other or only one is responsible for the liver lesions. The results of the present study help us to understand the biology of feed efficiency in cattle and drive our ongoing research on this important phenotype in animal science.

## Methods

### Phenotypic data collection

All animal protocols were approved by the Institutional Animal Care and Use Committee of Faculty of Food Engineering and Animal Sciences, University of São Paulo (FZEA-USP – protocol number 14.1.636.74.1). All procedures to collect phenotypes and biological samples were carried out at Pirassununga, State of São Paulo, Brazil. Ninety eight Nellore steers (16 to 20 months old and 376 ± 29 kg BW) were evaluated in a feeding trial carried out at FZEA-USP. Before the trial, the animals were kept in a single group and grazed primarily in *Brachiaria spp.* pastures. At the time of enrollment in the study, the animals were housed in individual pens or in group pens made of Calan Broadbent feeding doors (American Calan Inc., Northwood, NH, USA), with 25 m^2^ as a minimum space per animal. The test period was comprised of 21 days of adaptation to feedlot diet and place and a 70-day period of data collection. During the adaptation period, animals received corn silage (*ad libitum*), which was gradually replaced by an experimental diet (total mixed ration [TMR], 79.3 % TDN and 16.7 % CP in dry matter [DM] basis, Additional file 1: Table S7). In the experimental period, TMR was offered at 8:00 h and 16:00 h, so that 10 % refusals were allowed. The orts were weighed daily prior to the morning feed delivery to calculate the daily dry matter intake (DMI).

All animals were weighed at the beginning, the end and at every 14 days of the experimental period without fasting. Average daily gain (ADG) was computed as the slope of the linear regression of body weight (BW) on feeding days. Feed efficiency was estimated by feed conversion ratio (DMI/BWG), residual feed intake (RFI), residual body weight gain (RWG) and residual intake and body weight gain (RIG). RFI was calculated as the difference between the observed and expected DMI of the animal, predicted by regression equation as a function of average metabolic weight (MBW) and ADG [5]:

$$ DMI={\beta}_0+{\beta}_1\;ADG+{\beta}_2\;MB{W}^{0.75}+{\varepsilon}_1 $$

Where β_0_ is the intercept, β_1_ and β_2_ are the regression coefficients of the variables ADG and MBW^0.75^, respectively, and ε_1_ is the residue of the equation (i.e. RFI). RWG was calculated as the residue of the regression equation to predict ADG based on DMI and MBW:

$$ ADG={\beta}_0+{\beta}_1\;DMI+{\beta}_2\;MB{W}^{0.75}+{\varepsilon}_2 $$

Where β_0_ is the intercept, β_1_ and β_2_ are the regression coefficients of the variables DMI and MBW^0.75^, respectively, and ε_2_ is the residue of the equation (i.e. RWG).

RIG was calculated by the difference between RWG and RFI, as recently proposed by Berry and Crowley (2012) [6]. We performed all regressions using the PROC REG procedure from the statistical package SAS 9.3. Individuals presenting regressed ADG value greater or lesser than 2.5 standard deviation were removed from the analysis.

Cattle were ultrasound scanned by a trained technician using an Aloka 500 V real-time ultrasound on the 1st, 14th, 28th, 42th and 56th day of the feeding trial. The back fat thickness (BFT) and rib eye area (REA) were measured on the *Longissimus dorsi* muscle between the 12th and 13th ribs. Rump fat thickness (RFT) was measured on the *Biceps femoris* muscle.

The 40 animals selected as HFE and LFE groups (based on RIG) were slaughtered at the slaughterhouse of the FZEA-USP on two days with a 6-day interval. On those days data of live weight, hot carcass weight, pelvic and kidney fat weight and liver weight were collected.

### Sample collection and RNA extraction

At the end of the 70-day experimental period 16 animals, the 8 extreme animals from each group (HFE and LFE), were taken to a working chute in groups, not being without access to the diet for more than 2 h. Liver biopsies were carried out in accordance with Gröhn and Lindberg (1982) [62]. Samples were identified and immediately frozen in liquid nitrogen and then kept in a −80 °C freezer until RNA extraction.

RNeasy mini kit (QIAGEN, Crawley, West Sussex, UK) was used in accordance with guidelines supplied by the manufacturer to extract total RNA from around 30 mg liver biopsy samples. RNA quality and quantity were assessed using automated capillary gel electrophoresis on a Bioanalyzer 2100 with RNA 6000 Nano Labchips according to the manufacturer’s instructions (Agilent Technologies Ireland, Dublin, Ireland). Samples which presented an RNA integrity number (RIN) less than 8.0 were discarded and RNA was extracted again.

### Preparation and sequencing of Illumina RNA libraries

Preparation of libraries was carried out using a TruSeq™ RNA Sample Prep Kit in accordance with TruSeq® RNA Sample Preparation v2 Guide (Illumina, USA, 2012, Part # 15026495 Rev. D). Briefly, the mRNA was enriched from 1 μg of total RNA by two rounds of purification using oligo dT magnetic beads followed by fragmentation and cDNA synthesis by random hexamer primers and reverse transcriptase. Next, end repair and 3’ ends adenylation of the fragments was performed to prevent them to bind each other during the ligation of adapters. Bar-coded adapters were ligated to the cDNA fragments and a PCR reaction was performed to produce the sequencing libraries. The libraries were evaluated and quantified using Agilent 2100 Bioanalyzer and qPCR with KAPA Library Quantification kit (KAPA Biosystems, Foster City, USA). Adapter-ligated cDNA fragment libraries were run on Illumina HiSeq 2500 equipment using TruSeq PE Cluster Kit and TruSeq SBS Kit (2x100bp). Samples were sequenced in two lanes, each one containing 4 samples from the HFE group and 4 samples from the LFE group. An average of 27.7 million paired-end 100 bp reads was sequenced per sample.

### Reads alignment and differential expression analysis

Sequencing quality was evaluated by FastQC software (http://www.bioinformatics.babraham.ac.uk/projects/fastqc/). Poly A/T tails and adaptors were removed by Seqyclean software (https://github.com/ibest/seqyclean) and only reads with quality scores ≥ 20 and ≥ 50 bp length were kept for further analysis. TopHat 2.0.9. (Bowtie 2.1.0) was used to align each sample against reference genome *Bos taurus* UMD3.1, allowing two mismatches per read. Details of sequenced reads and mapping parameters for each library can be seen in Additional file 1: Table S8. Aligned reads were filtered using Samtools (−F 1792) [63] to remove secondary alignments, PCR duplicates and low quality alignments and then, read counts for each gene was estimated using HTSeq package [64]. Differential expression was performed using EdgeR package on R environment, which is based on the negative binomial distributions [65]. Gene expression was estimated as Counts Per Million (CPM) and were kept, for differential expression analysis, only genes which presented at least 1 CPM in at least 8 (half) samples. A hierarchical cluster analysis was performed to be sure that the differentially expressed genes (*P* < 0.2) were enough to differentiate de two FE groups (data not shown). Based on this analysis, one sample from each group was removed and the differential expression analysis was performed again with a total of 7 samples per group. Benjamini-Hochberg methodology was used to control the false discovery rate (FDR) and transcripts with Padj ≤ 0.1 were considered to be differentially expressed (DE).

### Gene co-expression network analysis

Gene co-expression network analysis was performed using the Weighted Gene Co-expression Network Analysis (WGCNA) R-package [66]. For this analysis, gene expression was estimated as quartile normalized Fragments Per Kilobases per Million reads (FPKM) using Cufflinks2 [67, 68]. As a quality control, genes which presented too many missing values (more than 7 samples with no counts) and genes with a mean > 0.5 and SD > 0.2 across the samples were excluded, considering that genes presenting low counts are less reliable and genes which vary little provide limited information in a co-expression analysis [69]. For computational reasons and because hub genes are expected to have more important biological functioning [70], from the 5299 genes which passed the quality control, we selected the 3500 most connected genes for further analysis. The measure of connectivity (K) is calculated by the sum of correlation between one gene and all other genes in the network. For these 3500 selected genes, an adjacency matrix was generated by calculating Pearson’s correlation between all genes and raising it to a power β (soft threshold) of 3, which is chosen using a scale-free topology criterion (R^2^ = 0.93). Next, to define cluster of genes based on degree of overlap in shared neighbors between genes, a topological overlap measure (TOM) was calculated and a value between 0 and 1 was assigned to each pair of genes. A value of 1 means that genes share the same neighbors and a value of 0 mean that they do not share any neighbor. The TOM matrix was then used as input to average linkage hierarchical clustering that results in a clustering tree (dendrogram) whose branches are identified for cutting, depending on their shape using the dynamic tree-cutting algorithm [71]. Modules containing at least 30 genes were detected and assigned to a color. Further information about the methodology and its relative merits can be found in [18, 70, 72].

In order to select potential biologically interesting modules for downstream analysis, Pearson’s correlation between the module eigengene and FE traits was calculated. The eigengene is the first principal component of a given module and a representative measure of genes expression profile in the module. A module was chosen for further analysis if it presented module-trait relationship > |0.5| for RFI or RIG (*P* ≤ 0.1). Furthermore, genes in selected modules were used for functional enrichment analysis only if their intra-modular connectivity with the module was >0.6 and the intra-modular connectivity with all other modules were <0.6. Intra-modular connectivity measures how co-expressed a given gene is with the other genes within the module and can also be called module membership.

Functional enrichment of GO terms was performed for each selected module using the online tool GOEAST [73], as it presents the results according to the hierarchy and relationship between the terms, facilitating the interpretation of results. Whole genome was used as background and P-values for each term were obtained through hypergeometric analysis and corrected for FDR by Benjamini–Hochberg method. Terms were considered significant when Padj ≤ 0.1. The same approach was used for the further enrichment analysis in this study.

### Detection of regulator genes

In order to detect regulator genes for the set of genes found to be related to FE traits by WGCNA, the Lemon-Tree software suit that uses probabilistic graphical models to identify modules of co-expressed genes and sign regulators from a previous selected list of genes was used [74]. For that purpose, the same 3500 genes selected as input for WGCNA were used. Expression data were first centered and scaled to have a mean of 0 and a standard deviation of 1. Then, the cluster algorithm was run 10 times to generate several clusters solutions that were next merged using the fuzzy clustering algorithm for a final robust cluster solution. To identify the regulator genes for each cluster we selected a list of genes from the input set based on their GO terms corresponding to signal transducer activity, kinase activity and transcription factor activity. This gave a range of 663 potential regulator genes that were assigned to nodes in the hierarchical tree by logistic regression generating probabilistic scores for each regulator. The significance of those probabilistic scores was calculated by a t-test of the mean of assigned regulators with the mean of randomly assigned regulators.

### Differential co-expression analysis

The aim of differential co-expression analysis is to uncover differences in modules and connectivity of genes between two sub-networks, one generated by expression data from 7 LFE animals and the other generated by expression data from 7 HFE animals. The set of genes for this analysis was the 5,299 genes which passed the quality control for co-expression analysis. Two sub-networks were generated by raising Pearson’s correlations between genes in LFE and HFE dataset to the power β = 20 and β = 4, respectively. Whole-network connectivity (K) was calculated for each condition and then a value of differential connectivity was assigned to all genes. Differential connectivity (K_Diff_) is defined as the difference of connectivity of one gene (i) in two different conditions (HFE and LFE) and was calculated first by dividing each gene connectivity by the maximum sub-network connectivity (K_HFE_ and K_LFE_) and then subtracting the values in one network from the other [16].

$$ KDiff(i)= KLFE(i)- KHFE(i) $$

This resulted in a normal distribution of values between −1 and 1 with negative values meaning that genes were more highly connected in the HFE sub-network than in the LFE sub-network and positive values meaning that genes were more highly connected in the LFE sub-network than in HFE sub-network. Genes presenting K_Diff_ > |0.6| were considered differentially connected and were used for functional enrichment analysis as described earlier.

### Serum biochemistry

Blood serum samples were collected on the 1st, 14th, 28th, 42th, 56th and 70th day of feeding trial. Serum biochemistry analysis was performed for the 20 animals from each group (HFE and LFE). Samples from day 1 and 70 were evaluated for total cholesterol, triglycerides, globulins, AST, ALT, GGT, ALP, albumin and total protein. Total cholesterol and GGT levels were also evaluated on samples from the 14th, 28th, 42th and 56th day.

### Histopathology

Immediately after slaughter, fragments of liver were collected for histopathological evaluation of 8 HFE animals and 8 LFE animals. Tissues were fixed in a 10 % formaldehyde solution for 48 h followed by histological processing according to routine techniques for inclusion in paraffin of our laboratory. Cuts of approximately 4 μm thickness were obtained and stained with hematoxylin and eosin (HE) [75]. Quantitative assessment was determined by percentage of affected portal spaces by mononuclear infiltrate in a 10x objective. All portal spaces present in histological sections were considered. The images were obtained by a Leica ICC50 HD microscope connected to a video camera system and computer using LAS EZ software.

### Statistical analysis

Sire effect on estimation of RFI and RIG were estimated by completely randomized design on SAS9.3 software using the following mathematical model:

$$ Yij=\upmu +\beta i+eij $$

where Y_ij_ is the observation of j^th^ individual, son of i^th^ sire; μ is the general mean of the trait (RFI/RIG); β_i_ is the sire effect; e_ij_ is the random residual error, ~NID(0,σ^2^_e_); and σ^2^_e_ is the residual variance.

The other statistical analyses were performed using GraphPad Prism 5.0 software. The phenotypic measures assessed in the HFE and LFE groups were first tested for normality by the Shapiro-Wilk test and later tested for difference between the means of the groups by Student's t-test for normally distributed data and Mann–Whitney-Wilcoxon test for nonparametric data. A two-way ANOVA followed by the Fisher post-test was used to test cholesterol and GGT levels on the different days of data collection (1st, 14th, 28th, 42th, 56th and 70th). The correlation between RFI and RIG was calculated by Pearson’s correlation. Results were considered significant when P ≤ 0.05 and tended to be significant when 0.05 < *P* ≤ 0.10.

## Availability of supporting data

The data sets supporting the results of this article are available in the ArrayExpress database (www.ebi.ac.uk/arrayexpress/) under accession number E-MTAB-3376, http://www.ebi.ac.uk/arrayexpress/experiments/E-MTAB-3376/.

## Additional files

Additional file 1:
**Supplementary tables.** (XLSX 108 kb) Additional file 2:
**Functional Enrichment of Brown Module.** Information contained in the boxes are the GO term accession number (i.g. GO:0006412), GO term name (i.g. Translation), the number of genes that contain the term per total input genes (i.g. 27/165), the number of genes in the genome that contain the term per total genes in the genome (i.g. 366/19926) and the adjusted *p*-value (i.g. 2.4e-14). (PDF 165 kb) Additional file 3:
**Functional Enrichment of Green Module.** Information contained in the boxes are the GO term accession number (i.g. GO:0006412), GO term name (i.g. Translation), the number of genes that contain the term per total input genes (i.g. 27/165), the number of genes in the genome that contain the term per total genes in the genome (i.g. 366/19926) and the adjusted p-value (i.g. 2.4e-14). (PDF 132 kb) Additional file 4:
**Functional Enrichment of Dark-orange Module.** Information contained in the boxes are the GO term accession number (i.g. GO:0006412), GO term name (i.g. Translation), the number of genes that contain the term per total input genes (i.g. 27/165), the number of genes in the genome that contain the term per total genes in the genome (i.g. 366/19926) and the adjusted p-value (i.g. 2.4e-14). (PDF 37 kb) Additional file 5:
**Functional Enrichment of Yellow Module.** Information contained in the boxes are the GO term accession number (i.g. GO:0006412), GO term name (i.g. Translation), the number of genes that contain the term per total input genes (i.g. 27/165), the number of genes in the genome that contain the term per total genes in the genome (i.g. 366/19926) and the adjusted p-value (i.g. 2.4e-14). (PDF 721 kb) Additional file 6:
**Functional Enrichment of Differential Co-Expression.** Information contained in the boxes are the GO term accession number (i.g. GO:0006412), GO term name (i.g. Translation), the number of genes that contain the term per total input genes (i.g. 27/165), the number of genes in the genome that contain the term per total genes in the genome (i.g. 366/19926) and the adjusted *p*-value (i.g. 2.4e-14). (PDF 924 kb)
