# Erratum to: Liver transcriptomic networks reveal main biological processes associated with feed efficiency in beef cattle

**DOI:** 10.1186/s12864-016-2649-7

**Published:** 2016-04-28

**Authors:** Pamela A. Alexandre, Lisette J. A. Kogelman, Miguel H. A. Santana, Danielle Passarelli, Lidia H. Pulz, Paulo Fantinato-Neto, Saulo Luz Silva, Paulo R. Leme, Ricardo F. Strefezzi, Luiz L. Coutinho, José B. S. Ferraz, Joanir P. Eler, Haja N. Kadarmideen, Heidge Fukumasu

**Affiliations:** Department of Veterinary Medicine, School of Animal Science and Food Engineering, University of Sao Paulo, Av. Duque de Caxias Norte, 225, Pirassununga, São Paulo 13635-900 Brazil; Department of Veterinary Clinical and Animal Sciences, Faculty of Health and Medical Sciences, University of Copenhagen, Copenhagen, Denmark; Department of Animal Sciences, School of Animal Science and Food Engineering, University of São Paulo, Pirassunung, Sao Paulo Brazil; Department of Animal Sciences, ESALQ, University of Sao Paulo, Piracicaba, Sao Paulo Brazil

## Erratum

After publication of the original article [1], it came to the authors’ attention that there were errors in the author list. The given name of **Joanir P. Eler** had been inadvertently misspelled, and **Saulo Luz Silva** was incorrectly listed as Paulo L. Silva.

The updated author list of the original article appears correctly in this erratum.
