# Supplementary figures and images for: Liver transcriptomic networks reveal main biological processes associated with feed efficiency in beef cattle
Source: BMC Genomics. 2015 Dec 18;16:1073. doi: 10.1186/s12864-015-2292-8 (PMC4683712; doi:10.1186/s12864-015-2292-8)

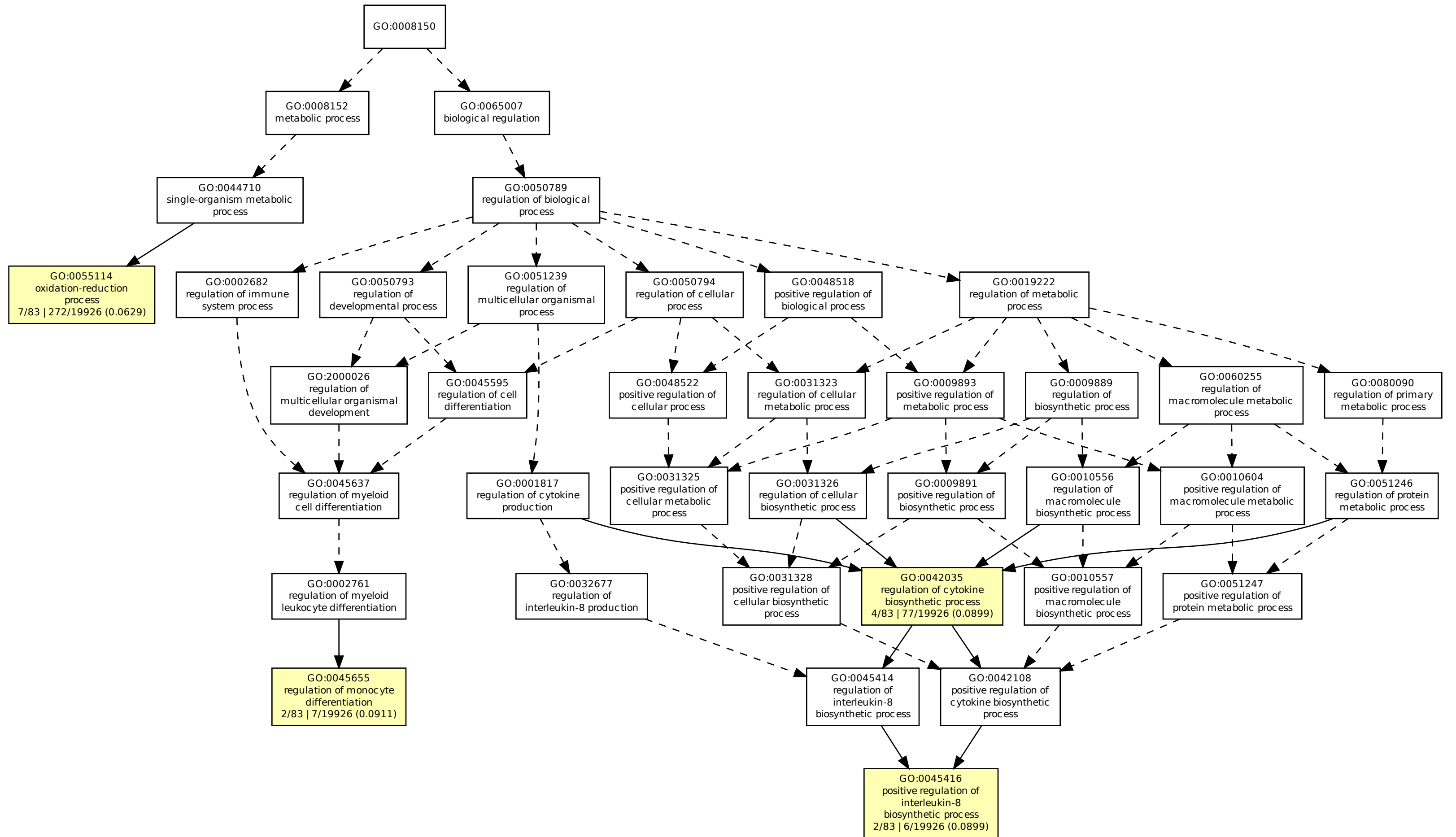

Supplement: Additional file 2: — Functional Enrichment of Brown Module. Information contained in the boxes are the GO term accession number (i.g. GO:0006412), GO term name (i.g. Translation), the number of genes that contain the term per total input genes (i.g. 27/165), the number of genes in the genome that contain the term per total genes in the genome (i.g. 366/19926) and the adjusted p-value (i.g. 2.4e-14). (PDF 165 kb) [file 12864_2015_2292_MOESM2_ESM.pdf]

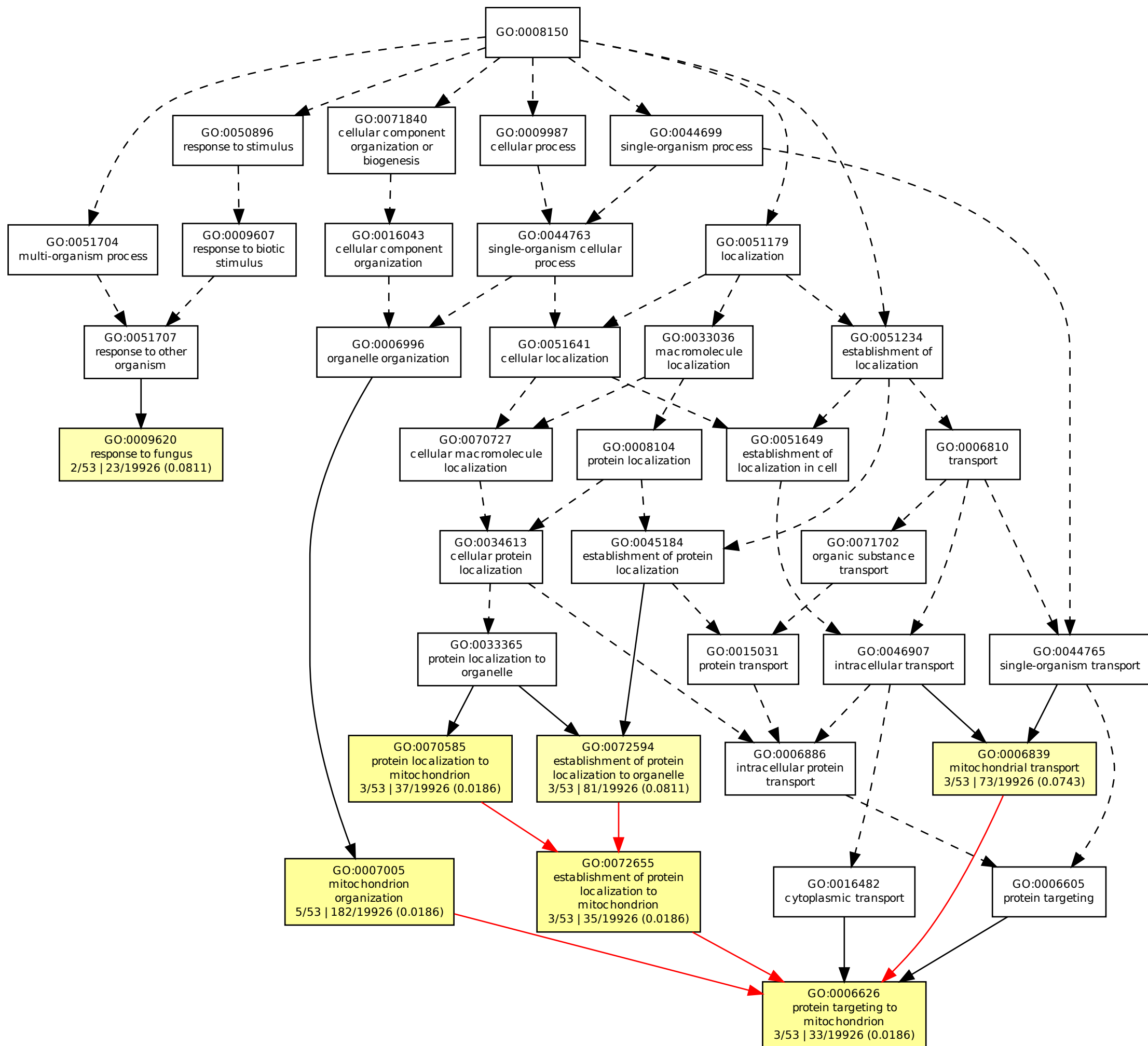

Supplement: Additional file 3: — Functional Enrichment of Green Module. Information contained in the boxes are the GO term accession number (i.g. GO:0006412), GO term name (i.g. Translation), the number of genes that contain the term per total input genes (i.g. 27/165), the number of genes in the genome that contain the term per total genes in the genome (i.g. 366/19926) and the adjusted p-value (i.g. 2.4e-14). (PDF 132 kb) [file 12864_2015_2292_MOESM3_ESM.pdf]

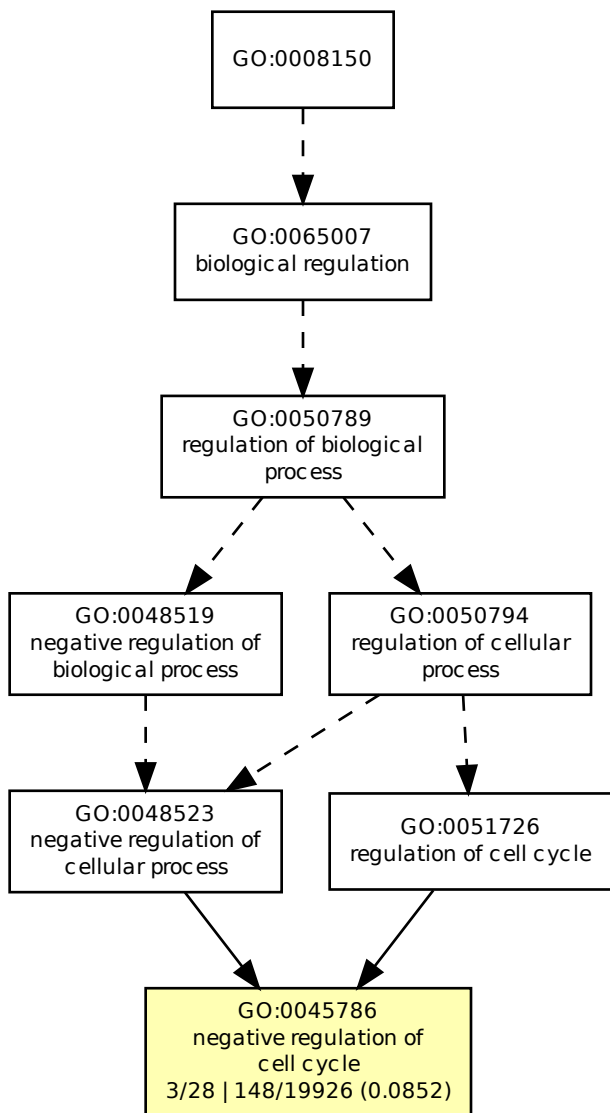

Supplement: Additional file 4: — Functional Enrichment of Dark-orange Module. Information contained in the boxes are the GO term accession number (i.g. GO:0006412), GO term name (i.g. Translation), the number of genes that contain the term per total input genes (i.g. 27/165), the number of genes in the genome that contain the term per total genes in the genome (i.g. 366/19926) and the adjusted p-value (i.g. 2.4e-14). (PDF 37 kb) [file 12864_2015_2292_MOESM4_ESM.pdf]

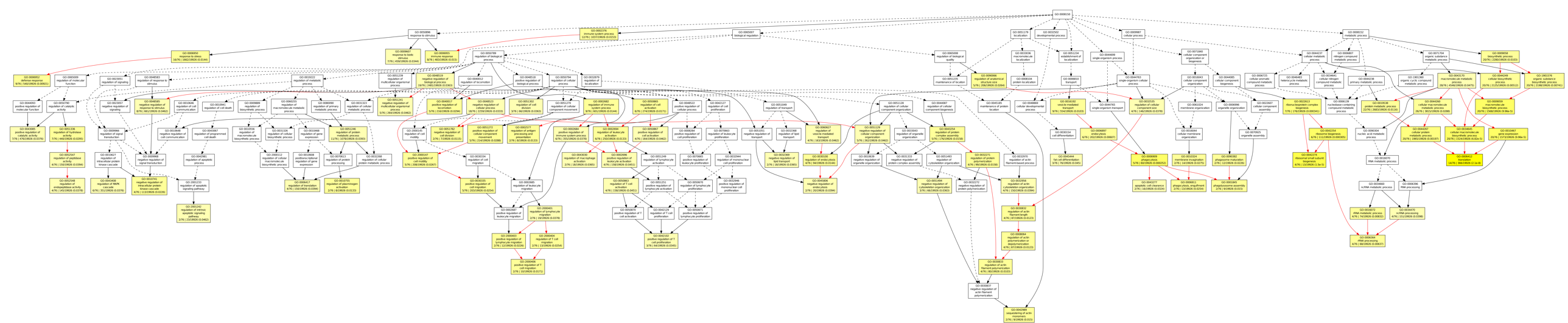

Supplement: Additional file 5: — Functional Enrichment of Yellow Module. Information contained in the boxes are the GO term accession number (i.g. GO:0006412), GO term name (i.g. Translation), the number of genes that contain the term per total input genes (i.g. 27/165), the number of genes in the genome that contain the term per total genes in the genome (i.g. 366/19926) and the adjusted p-value (i.g. 2.4e-14). (PDF 721 kb) [file 12864_2015_2292_MOESM5_ESM.pdf]
